# Analysis of Microbial Communities: An Emerging Tool in Forensic Sciences

**DOI:** 10.3390/diagnostics12010001

**Published:** 2021-12-21

**Authors:** Audrey Gouello, Catherine Dunyach-Remy, Christian Siatka, Jean-Philippe Lavigne

**Affiliations:** 1 Institut de Recherche Criminelle de la Gendarmerie Nationale, 95037 Cergy-Pontoise, France; gouello.audrey@outlook.fr; 2 Bacterial Infection and Chronic Infection, INSERM U1047, Department of Microbiology and Hospital Infection, University Hospital Nîmes, Université de Montpellier, 30908 Nimes, France; catherine.remy@chu-nimes.fr; 3 Ecole de l’ADN, Université Nîmes, 30021 Nimes, France; christian.siatka@unimes.fr

**Keywords:** body fluid determination, criminalistic, forensic sciences, human identification, microbial communities, skin microbiota

## Abstract

The objective of forensic sciences is to find clues in a crime scene in order to reconstruct the scenario. Classical samples include DNA or fingerprints, but both have inherent limitations and can be uninformative. Another type of sample has emerged recently in the form of the microbiome. Supported by the Human Microbiome Project, the characteristics of the microbial communities provide real potential in forensics. They are highly specific and can be used to differentiate and classify the originating body site of a human biological trace. Skin microbiota is also highly specific and different between individuals, leading to its possibility as an identification tool. By extension, the possibilities of the microbial communities to be deposited on everyday objects has also been explored. Other uses include the determination of the post-mortem interval or the analysis of soil communities. One challenge is that the microbiome changes over time and can be influenced by many environmental and lifestyle factors. This review offers an overview of the main methods and applications to demonstrate the benefit of the microbiome to provide forensically relevant information.

## 1. Introduction

**Figure 1:**
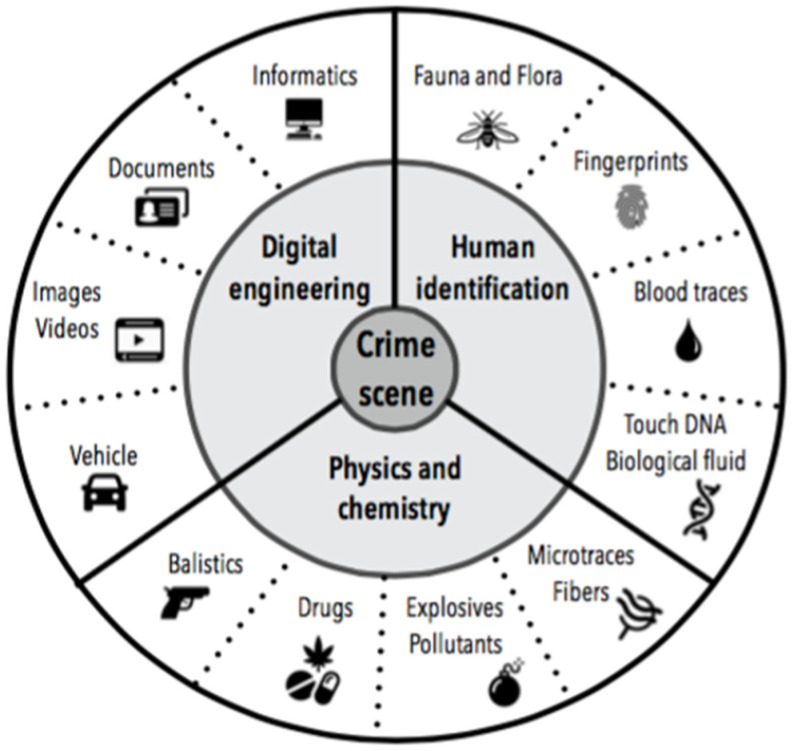
Some clues that can be recovered at a crime scene.

Trace evidence is commonly defined as the surviving evidence of a former occurrence or action of some event or agent [1]. Analysis of the various materials present on a crime scene is an important part of proceedings, with the potential to reveal presence or connections between individuals or with an object or a place. These marks of criminal action are commonly classified into three major components: digital engineering, physics and chemistry, and human identification (Figure 1). Alphonse Bertillon, a famous French scientific forensic police officer, was the first to use microorganisms as trace evidence and to determine their origin site. He analyzed about 500,000 microbes/m^3^ and 88,000 microbes in various sites in Paris [2].

Any object can be used as evidence if it can be related to the crime scene. Physical evidence is often crucial for criminal investigations to complete victim, suspect, or witness testimony [3]. DNA and fingerprints have the advantage that they can be collected on many different supports. In forensic science, production, sampling, and analysis of traces have been modelled by Inman and Rudin with six main notions: divisibility, transfer, classification, identification, association, and reconstruction [4]. 

**Figure 2:**
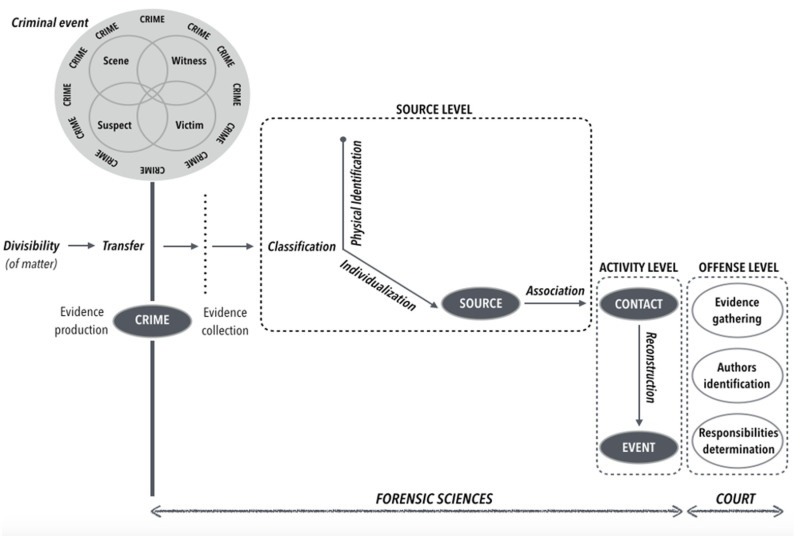
Principles of forensic sciences (adapted from [4]).

There are three levels of crime scene analysis: the source, the activity, and the offense. The source level consists in making proposals on the material origin of the trace to determine an individual profile (what is the donor of the biological trace? who was present on the crime scene?). The activity consists of formulating hypotheses on the mechanisms and/or actions having led to the trace deposit to reconstruct the crime scene (what was the sequence of events?). Finally, the offense, in magistrates’ hands, consists in evaluating the criminal action as a whole, about the relevance of traces and elements of the context or intention (who is guilty? who committed the crime? and why?) (Figure 2). 

Traditionally, both DNA analysis and fingerprints are used to identify a suspect, but these techniques have some limitations: DNA can be degraded, contaminated, or in too low amounts to have interpretable results; fingerprints can be partial or overlapping [5]. Moreover, body fluids traces are generally available in low quantities and may be in mixtures; so their identification could be difficult [6].

To reconstruct the crime scene chronology and events, it is important to determine the presence and origin of biological evidence. Current genetic analysis based on Short Tandem Repeat (STR) sequences establishes an individual genetic profile, but they cannot determine how the trace was deposited [7]. The determination of the origin of biological fluids can help to answer this question. To identify biological fluids like saliva, blood, or sperm, some presumptive or confirmatory tests are currently used, whether chemical or immunological. However, these tests are limited in sensitivity and/or specificity [6,8]. Other alternatives are applied to body fluid prediction and differentiation such as mRNA, miRNA, or epigenetic markers like methylation degree of GpC islands [7]. Several markers have been identified for common forensic biological fluids such as ankyrin 1 for blood, protamines for semen, or histatin for saliva [6,9]. They are also easily recovered from many surfaces and are relatively stable in body fluids. The principle is to detect the transcripts number for each body fluid by measuring the total DNA or RNA, but these methods lack specificity. Moreover, these mRNA markers are influenced by many factors (e.g., age, diet, health). miRNA appears to be a good alternative because they are very stable, even in post-mortem samples, and some are human specific. However, a disadvantage of miRNA markers is that they are not very specific to a body part or fluid. Several studies have nevertheless determined some specific markers for peripheral blood, semen, and vaginal epithelium [10]. Finally, the epigenetic studies are based on the DNA methylation analysis. Epigenetic differences on GpC islands are observed between biological fluids and can be detected by PCR and/or bisulfite conversion [6,9]. Their use in combination seems to be the best alternative [7].

The emergence of next generation sequencing (NGS) technologies that combine miniaturization and performance allowed to considerably reduce the time needed to sequence one or more genomes. They make it possible to identify the source of single or multiple traces but also to bring additional information to the investigation. Despite these advances in DNA analysis, many criminal cases remain unsolved to date and the need to improve proof elements is essential to connect the perpetrator to the crime scene [11]. These NGS technologies can be applied to the study of the human microbiota on different organs. Thus, the emergence of microbiota analysis represents a promising alternative to the current method for forensic analysis because bacteria are ubiquitously present in all parts of body, but each individual has their own microbiota.

## 2. Use of Microbiome in Forensic Sciences

One of the biggest advantages of microbiome analysis used in forensics is the diversity and the ubiquity of the microbial community in each person. Indeed, the human microbiota consists of 10–100 trillion symbiotic microbial cells unique to an individual [11,12,13]. Studies have shown that the human microbiome consists of various bacterial communities at different body sites. For example, there are about 200 species in the mouth and about 400 species in the intestinal tract [14]. Moreover, bacterial DNA is less sensitive to degradation than human DNA due to its circular form and its membrane. Thus, techniques used in medical analysis can be applied to crime scenes and provide more information than DNA alone. Currently, there is an explosion of microbiome analysis in medicine and public health, ecology, and industrial applications, but its use in forensics is an emerging tool [15,16,17,18] and is limited in criminal investigations [19]. 

The human skin is composed by a large cutaneous microbiota that can be deposited and disseminated easily on many surfaces or objects [20,21]. These microbial communities can remain on touched surfaces for a long period because bacteria have high resistance to environmental stress like moisture, temperature, or ultraviolet radiation. The individual’s lifestyle influences the microbiome composition (e.g., food, life condition, health status, consumption of cigarettes, living with pets). Thus, humans and environmental samples are in constant interaction involving microbial exchanges. It is possible to recover specific bacteria from classrooms, rooms, and offices to demonstrate the presence of an individual [21,22]. The characterization of the personal microbiome could identify a suspect leaving their bacterial community at the crime scene or directly on the victim [22]. 

The principle of microbial forensics is based on characterizing the microorganisms in a sample, by determining the association between the presence of bacterial communities and the concerned body site or tissue. The first case was a biological attack with *Bacillus anthracis* in the United States, 1 week after the 11 September terrorist attack. This species was sealed in envelopes and delivered to American senators and media offices. The Federal Bureau of Investigation (FBI) undertook microbial forensic investigations to identify the perpetrator. The investigation lasted several years because the analysis techniques were not yet adapted, particularly the availability of reference strains and the evaluation of the evidence value. The FBI officially ended its investigation in 2011 [16,23]. In this example, microbial forensic was mainly applied to isolate pathogens used as biological weapons or trace transmission of some human viruses between criminals [21]. Preliminary microbial studies in forensics have determined the presence or absence of bacterial species to define specific markers. Six specific microbial markers were highlighted to distinguish vaginal, oral, and fecal samples [24]. However, these studies used very few markers and samples, and are therefore not representative of reality and many false positives or negatives were observed.

**Figure 3:**
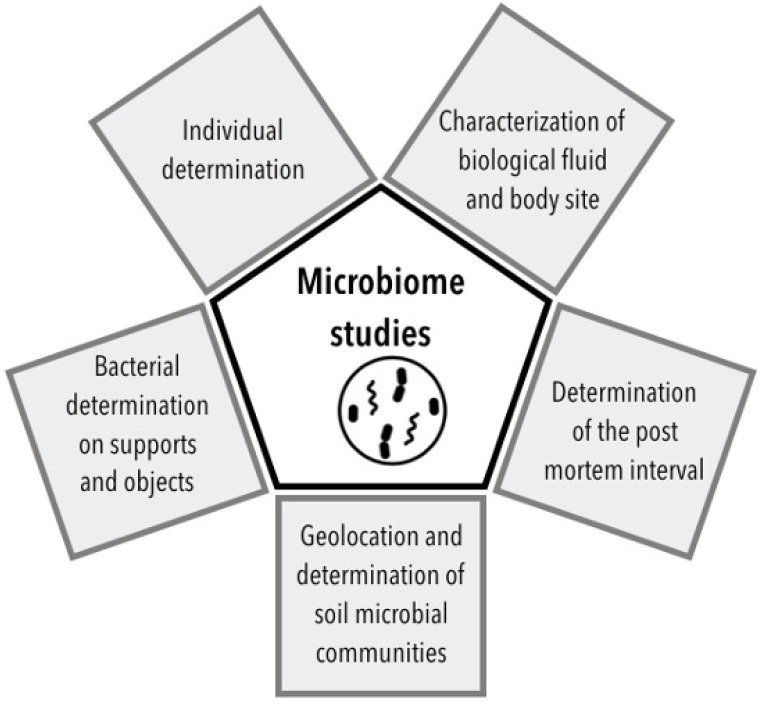
Microbiome analysis toolbox.

Since then, many recent studies have explored the possibilities of microbiome analyses. This review is an overview of the main techniques and results obtained for microbial communities’ explorations (Figure 3).

## 3. Microbiome Analysis and Individual Determination

The composition of the microbiome in the different parts of the body seems to be unique and stable over time [13]. The skin represents the first line of defense between pathogens and the body. However, this barrier is not sterile and many microorganisms (about 10^7^ cells/cm^2^) live together, composed mainly by bacteria but also viruses, fungi, and dust mites [25]. Thus, the skin bacterial communities are diverse, dynamic, and vary in each part of the skin coating [26]. For example, in the palm of the hand, only 13% of bacteria are shared between individuals [20]. Skin homeostasis is notably maintained by the bacterial communities, but the combination with other less abundant species explains that the cutaneous microbiota is unique [27]. Interestingly, skin communities vary much more than those of the gut or oral cavity, because they are more exposed to environmental changes [28].

### 3.1. Determination of the Core Microbiome

Although each individual has a unique bacterial profile, there are similarities between individuals, represented by the core microbiome. It corresponds to the bacterial communities shared by all individuals and undoubtedly contributes to essential functions in the human body [29]. Schmedes et al. worked on the association of various skin sites with their individual host by identifying specific bacterial markers [30]. Ten major bacterial species were relevant for forensic analyses, in particular *Cutibacterium* (formerly *Propionibacterium*) *acnes*, which was identified in all samples. Its presence/absence could be a criterion of choice to determine if the collected sample corresponds to a skin sample. However, this study was not conducted under forensic conditions [30]. The authors completed their study by using hidskinPlex markers (common and individual markers) to differentiate skin microbiomes from different individuals. This new sequencing method seems to be promising because it can distinguish different body sites [31]. 

Costello et al. studied the variation of microbial communities across different body sites by sampling diverse skin locations [28]. Each body site had its own specific and stable core microbiome between people over time. However, the inter-individual variations were greater than the intra-individual variations, and the evaluation of the core microbiome was dependent on the examined body site.

### 3.2. Example of Specific Bacterial Communities

Hands play an important role in forensic investigation. Studies have evaluated the “normal” composition of this microbiome and how it could vary and be transferred to a support [32]. Hands represent a specific microbiome composed of 8–24 main families of bacteria and about 150 species. *Micrococcus* and *Staphylococcus* species were the main species identified [33]. However, while a fingerprint is stable over time, the microbiota recovered on hands are subject to many changes and are therefore less stable. Intrinsic and extrinsic physiological conditions of hands influence the stability of this microbiome. Age and sex have an impact on microbial composition. Firmicutes species were abundant on children’s hands, while more *Propionibacteria* were identified in adults. Moreover, women’s hands presented a greater bacterial diversity. This observation could be linked to the male skin pH, which is more acidic, or linked to hormonal characteristics of hands [25,34]. Further studies are needed to evaluate the persistence of microbial communities on hands [35]. 

### 3.3. Personal Microbial Cloud

Based on the principle of a continuous exchange between an individual and the surrounding environment, Meadow et al. studied what they called the “personal microbial cloud” to determine a human microbiome signal. This cloud corresponds to microbial communities around an individual, like a personal aerosol. In a sterile room, with controlled conditions (pressure, temperature, air filtration) in which individuals had been placed for several hours, they collected air filters and identified human and individual specific microbial communities. Each participant left an identifiable microbial signal. Then, they collected air surrounding the participants and found that airborne particles were directly linked to them. Despite limitations to this methodology including the number of participants, the amount of analyzed particles, the ambient conditions, the time spent in the room, and the microbial background of the room, this approach is interesting to evaluate the skin microbiota in a controlled environment [36]. 

### 3.4. Cross Transfer between Individuals

Studies have investigated the interaction between human microbiome and personal items. In forensic investigations, analysis must define if this transfer was direct (human-object) or indirect (human-object-human). Human microbial communities can be a mixture of many microorganisms. Do they come from a single source? Is there a transfer from an object or another human? Or from another body site? Neckovic et al. studied direct transfer (following a handshake) and indirect transfer through an intermediary substrate (previous touched object or surface) [37]. Over 3 days, they sampled paper, cotton fabric, and glass after they were handled by different non-cohabitating individuals, and a reference sample was done on the right finger on each individual. They demonstrated that both transfers had an influence on the microbial communities recovered. The difficulties were to materialize this transfer and determine its persistence over time. This calls into question the possibility of connecting an individual to an object. However, it could be interesting in cases of sexual assault where there is strong skin-to-skin contact. To apply this method to forensic science, it is necessary to avoid contamination risks [37].

## 4. Interest in the Study of Biological Fluids Microbiomes

Genetic STR polymorphisms analyses establish an individual’s genetic profile, but they do not determine how traces are deposited on the site. The characterization of body fluids is a key step in forensic investigations and can help to understand what happened at the crime scene. For example, in the case of a bite, it would be interesting to find the suspect’s saliva or in the case of supposed sexual assault, it would be necessary to define vaginal microbial communities. The spatial positioning of such biological samples can imply consent/non-consent of sexual relationships and support the testimony of victims or suspects.

### 4.1. Saliva

Saliva is a common biological fluid, which can be recovered from many supports in a crime scene, arising from kissing, biting, or licking. It contains approximately 500 million bacterial cells/mL and around 700 different bacterial species [17]. The oral cavity is the first entry of microorganisms into the digestive system, and teeth, tongue, and cheek promote microbial colonization. As the mouth is regularly exposed to the external environment, a dynamic and quite high variability in the oral cavity is observed [38]. The oral microbiota can be influenced by factors such as tobacco, drugs, or cosmetic products. Few studies explore the stability and the dynamic of the oral microbiome over time. However, a saliva core microbiota was described, suggesting a certain individual stability [38]. This stability can exist over a shorter period but varies over a long-term period [38]. 

Jung et al. developed a new tool for saliva identification based on multiplex real time-PCR, using three oral bacterial markers [39]. This method was specific (only one saliva marker was detected in one feces sample) and sensitive (the detection of markers was possible with low quantity of saliva until 14 weeks. These specific oral bacteria were identified in 91.4% samples and DNA levels at very small concentrations (0.13 ng of total DNA). The authors observed that tooth brushing had a negligible effect on the presence of the specific markers. Indeed, tooth brushing slightly impaired microbial community detection on cigarettes, mugs, forks, or bite marks, but all samples were considered positive. Moreover, a long storage period was compatible with the detection of markers. Finally, no cell lysis treatment is necessary, allowing the sample to be further analyzed to determine a genetic profile of the suspect [39]. 

Leake et al. analyzed the inter- and intra-individual variations of saliva [17]. Saliva from two healthy adults was collected at different points and three targets were used to improve the identification (the *rpoB* gene 1 (*Streptococcus*) and 2 (other), and the 16S rRNA). They demonstrated that the salivary microbiota was specific, and this approach distinguished two individuals [17]. The number of volunteers was too small in this study and a larger study must be done to corroborate the value of this tool. 

### 4.2. Vaginal Secretions

Vaginal fluids identification is essential during sexual assaults. Among these secretions, *Lactobacillus* species are the main dominant communities in human healthy vaginal secretions, they protect women against pathogens and opportunistic infections [13,40]. Microbial composition is relatively stable in vaginal samples, regardless of the female menstrual cycle stage or pregnancy. Diseases such as vaginosis could have an impact on the “normal” vaginal microbiota and create dysbiosis [41,42].

Giampaoli et al. analyzed vaginal, oral, and fecal swabs, and some forensic samples from female genital regions by real-time PCR [24]. Results were coherent with the body site, although some vaginal bacteria were also present in fecal samples. They also studied mixtures of different samples and observed no influence on typical vaginal flora and the possible interpretation of these complex samples. These promising results suggest the ability to distinguish individuals using microbiota approaches in case of sexual assault or violence. Moreover, the authors used the same extraction protocol for microbial communities and STR analysis, avoiding the need to obtain multiple samples during forensic investigations [24].

Doi et al. developed a method to identify *Lactobacillus* in vaginal secretions using real-time PCR [43]. They designed a *Lactobacillus* primer by using sequences alignment of 16S RNA from various *Lactobacillus* species. This tool discriminated vaginal secretions (with low cycle threshold (Ct) values) and other body fluids. 

### 4.3. Blood Samples

Diez Lopez et al. determined a classification of blood, a common fluid found in crime scenes, using 16S rRNA gene sequencing [44]. They distinguished four types of blood: menstrual blood, venous blood, nasal blood, and blood from skin epithelium. As expected, menstrual blood samples were easily classified by the dominance of *Lactobacillus* species although the day of the menstrual cycle and possibly vaginal diseases can affect the results. For venous blood, non-specific products were characterized corresponding to human host proteins. Low quantities of bacteria were detected due to the sterile nature of blood in a healthy patient. Nasal blood results were more poorly conclusive due to the importance of the nasal blowing that could dilute microorganisms. However, the presence of bacteria from nasal microbiota suggested the origin of the blood. Similarly, the detection of microorganisms on cutaneous microbiota helped distinguish blood from skin epithelium [44]. This taxonomic screening method must be evaluated in larger studies.

### 4.4. Fecal Material

Fecal samples are representative of the gut microbiota. Contrary to other samples, the quantities of bacteria are particularly high. However, exposure of the largely anaerobic microorganisms to aerobic conditions could pose difficulties to determining the microbiota. The gut microbiota is classically composed by two main phyla, *Firmicutes* and *Bacteroidetes* [45]. These bacterial communities are relatively stable [21], forming the gut core microbiome. However, a high degree of variation between individuals has been observed at the species scale and microbiome analysis could, for example, distinguish monozygotic twins [46]. The final diversity of gut microbiota is established around 4 years old. This composition could vary according to many parameters such as genetics, health, age, lifestyle, diet, environmental conditions, or exposure to treatments. For example, antibiotics had a particular influence on gut microbial communities [42,46,47]. The gut microbiota is considered as the equivalent of the digital print. 

### 4.5. Scalp and Pubic Hairs

Hair is easily recovered from crime scene, but there is often too little nuclear DNA to establish robust genetic profiles. Hair shaft supports specific and individual microbial communities [26]. Tridico et al. applied metagenomic approaches on hair analysis by focusing on three factors: the differentiation between scalp and pubic hairs by microbial composition, the differentiation between individuals by colonizing microorganisms, and the persistence of hair bacterial communities over time [48]. The pubic hair microbiota was relatively stable and less affected by environmental bacteria than the scalp hair one; however, sexual intercourse can modify these populations. As for vaginal secretion, *Lactobacillus* sp. was most frequently recovered in female pubic hair, acting as a barrier for other microorganisms. This species allowed the distinction between female and male pubic hairs. No significant difference between male and female microbial communities was noted on scalp hairs, but this microbiota seemed to be more sensitive to environmental fluctuations than pubic hairs [26,48]. Interestingly, Brinkac et al. observed that geographical origin (different states in USA) influenced the composition of hair microbiota, making this type of sample a good forensic candidate. The authors also noted that the characteristics of hair itself, especially its length, had an impact on the microbial diversity found [26].

Williams et al. analyzed samples from the pubic hairs of sexually active couples. They determined a majority of *Corynebacterium* species in male samples and *Lactobacillus* species in female samples and highlighted a mix of microbial communities in case of sexual activity. The similarity between individual microbial communities increased with the sexual relationship frequency. They also showed that couples shared hair microbiome (pubic, ears) but there was no correlation with sexual activity. The potential transfer and the link between victim and assailant were also investigated. After creating a scenario with a victim, her partner, and an unknown assailant, Williams et al. generated a new microbial profile corresponding to the assailant, but the formal identification of this individual was clearly impossible. However, this technique could be interesting for narrowing the field of potential suspects [49].

### 4.6. Sperm Sample

The testicular sperm microbiome is composed of a low biomass but with abundant contamination [50], whereas the semen microbiome is rich and diverse [51]. Baud et al. studied the composition of the seminal fluid and its impact on sperm parameters [52]. Recovered microbial communities were sperm-specific. *Lactobacillus* and *Staphylococcus* genera were associated with normal sperm, similar to vaginal and cutaneous microbiota. The composition of this microbiota had no influence on sperm quality. However, the presence of pathogenic bacteria (e.g., *Ureaplasma urealyticum*, *Mycoplasma hominis*, and *Prevotella* sp.) negatively affected the quality of semen [51]. This parameter could be promising in forensic studies for solving cases where no spermatozoids are observed [52]. 

### 4.7. Other Samples

Quaak et al. investigated other supports such as mouth, vagina, feces, penis, and hand samples to differentiate these microbial communities [53]. Oral and stool samples had a higher microbial diversity than vaginal or skin communities. *Lactobacillus* species and *Streptococcus salivarius* were specific to vaginal and oral microbiota, respectively. However, these species have also been recovered in other samples. Interestingly, the amount of each species was specific enough to determine the body site origin [53]. The knowledge of the core microbiome for each body fluid or body site seems to be the most important preliminary result in forensic investigations. 

## 5. Microbiome Determination on Supports and Objects

Trace DNA from supports like glasses, phones, and door handles can be used in microbial studies [22]. This relationship between the bacteria from human cutaneous microbiome and the microbiome of surfaces or objects highlights how the human microbiome can shape the microbial ecology of different locations (e.g., homes, offices, and cities). Although the dynamic changes of the microbiome have been studied, less attention is paid to the stability of external human samples. These studies may be important in forensics to predict the source of the human body fluids because samples from crime scenes may be exposed to natural human habitats, storage conditions, and lab analysis [8,19].

### 5.1. Mobile Phones

Mobiles are often used daily and can provide information such as location or health indicators. Many studies have demonstrated that microbial communities from skin could be recovered on phones. Samples from the thumb, fingers of the dominant hand, and smartphones touchscreen showed bacterial communities were linked to human body habitats. Interestingly, a marked difference could be observed between men and women; the composition of the bacterial community of fingers of females was very similar to their phones, unlike men where it was different [54]. Lax et al. demonstrated that microorganisms recovered from the back of phones corresponded to microbial communities of hands, whereas microorganisms on the front of phones belonged to face microbiota. Moreover, microbial communities on phones were not stable, notably due to the hyper-volatility of the microorganisms present on the owner’s hands. It is important to note that samples were recovered less than 3 days after their deposit, making this investigation difficult to apply to forensic sciences [55].

### 5.2. Keyboards and Mice

Fierer et al. evaluated the microbial communities recovered from computer keyboards and mice with those of the users [20]. The bacterial communities were similar on computer keyboards or mice and fingers of the owner, suggesting that the individuals possess a unique bacterial “fingerprint”. The influence of the storage conditions on the objects’ microbial communities was minimal on bacterial community composition. 

### 5.3. Shoes

To determine the influence of geography on microbial communities, Lax et al. recovered samples on shoes of three participants living in different towns in the USA and Canada in parallel with soil samples [55]. They highlighted a significant difference between the participants’ shoes, likely due to the core microbiome of individual shoes. Unsurprisingly, the majority of the microorganisms recovered from shoes came from soil. Inversely, shoe microbial communities could be deposited on soil and influence the floor bacterial communities. The transfer of microorganisms seemed to be independent of the floor surface material. 

### 5.4. Identification of Objects Handled by the Decedent before Death

Kodama et al. screened microbial communities to identify which objects had been manipulated by a victim before death [19]. The authors compared cadaver skin microbiomes (on the right palm) and 100 potentially handled objects (such as phones or lighters) at 16 different crime scenes. Post-mortem skin microbiomes appeared to be stable during morgue transport and storage. This information could help estimate the post-mortem interval (PMI) and would allow recovery of microorganisms directly on the cadaver and not only at the crime scene. However, it is impossible to date when the victim had touched the objects. 

## 6. Determination of the Post-Mortem Interval (PMI) Using Microbiome Analysis

Estimating PMI is a well-documented application of microbial community investigation. PMI can be essential to estimate the death time, or write death certificates. PMI is often difficult to establish because death mechanisms are very complex [3]. After death, the body undergoes irreversible, gradual physical and chemical changes [56]. This organic matter decomposition, especially cadaver decomposition, engages biological factors like cell enzymes, bacteria, fungi, protozoa, insects or carnivores, and non-biological factors linked to the environment such as humidity or weather [57]. Insects, invertebrates, and other organic elements all participate in the decomposition process, in a structured order [58]. The PMI is traditionally estimated by physical changes occurring after death, like rigor mortis, lividity, temperature, or upper gastrointestinal activities [57,59]. However, these changes take place at the beginning of the post-mortem process, leading to a rough estimate of PMI. Other recent techniques to estimate the PMI include thanatochemistry, DNA/RNA degradation, and entomology. The study of insects and their activities can be inhibited by environmental conditions such as rain, low temperature, indoor environments (where no or few insects are present), and seasons (when insect activity is low) [57,59]. Other approaches must be developed.

Recently, microbial communities’ changes have been evaluated to estimate the PMI and the circumstances of death [5,12,60]. Metcalf et al. observed that the microbial communities changed with each step of cadaver decomposition, defining a “microbial clock” [3,12,60,61]. Bacteria recovered in internal organs are directly linked to the decomposition and 75–90% of cells before death are microbial cells [62,63]. Two methods are used for microbiome evolution during decomposition: (i) establishing the thanatomicrobiome, which is the study of microorganisms found in internal organs and cavity after death; and (ii) the epinecrotic communities, which consists in studying prokaryotes, protists, fungi, and eukaryotes living at the human surfaces [62,63]. 

The microbial community succession is relatively repeatable and predictable in each individual. Commensal bacteria play a role in metabolizing cellular products resulting from cells lysis due to the lack of oxygen and are considered a major component of cadaver decomposition. After death, internal bacteria begin to digest the body from the inside, and dead cell enzymes destroy tissues [57,64]. The loss of biomass is mainly driven by insects and microorganisms [57]. Bacterial communities have two distinct steps in the decomposition process [3,65]. Various anaerobes grow in the abdominal cavity for the first 9 days. These microbial activities lead to gas development in the different body parts and the liquefaction of tissues. Next, oxygen access leads to aerobes and opportunistic pathogens development, and particularly, the increase of pH creating favorable conditions for *Proteobacteria*. During decomposition, environmental conditions create an imbalance of common bacteria and allow opportunistic pathogens to settle and grow [66]. The bacterial community of the cadaver changes to reflect the surrounding environment. By studying grave soil, abdominal cavity, and skin microbiomes, the succession of these specific bacteria allows estimation of the PMI [57]. Metcalf et al. were able to accurately estimate the time since death to within approximately 3 days [3,65]. Combining the 16S and 18S rDNA analysis gave the best estimate of the PMI. Nevertheless, some inter-individual variability in the succession of bacterial communities could not be excluded [58].

Many environmental factors such as temperature, grave soil, humidity, oxygen, precipitation, and presence of insects or scavenger animals can influence this sequencing decomposition of a microbiome [56,59]. The soil decomposition is linked to the microbial activity. During corpse decomposition, the soil chemistry changes, especially organic carbon, ammonia, and nitrogen [58]. Hyde et al. tracked two similar cadaver decompositions over roughlt 2 weeks (in September and November). The environmental conditions surrounding the cadaver influenced the bacterial communities present and the decomposition steps. Some differences had been also observed in the microbial composition and structure between body sites [62,64]. Guo et al. confirmed that many factors influence results, such as the first microbial communities establishing in and on the cadaver, the environment of the cadaver, and the sample collection method [57]. Interestingly, they also demonstrated that the presence or absence of sarcosaphagous insects had no significant impact on microbial community taxa, eliminating one environmental factor [57].

The microbial clock is most accurate within 48 h of discovery, when the cadaver is fresh, and not yet contaminated by soil microorganisms. After this, the error risk increases with time. Indeed, for corpses in advanced decomposition stage, predicted PMI carries an error rate of about 5–7 days. If only the skeleton remains, PMI can be determined from microbes present in bones. The body conditions (e.g., age, cause of death, underlying pathologies, consumption of drugs, diet) can influence the estimation and must be considered in the calculation [59]. Pechal et al. demonstrated that sex and organ type had a significant influence on PMI determination, while location, season of death, and weight did not [64]. Death conditions also seemed to have an impact on recovered bacteria: heart diseases had a lower diversity, while violent deaths presented a more substantial microbial diversity [67].

Finally, proteomic approaches have been evaluated. Although the majority of bacterial communities have been detected by MALDI-ToF analysis, many false positives have been observed due to high peptide signal in muscles, requiring additional developments [66]. 

## 7. Geolocalisation and Determination of Soil Microbial Communities

Microbial communities are different depending on where samples are recovered. Indeed, bacterial composition varies with geographical locations according to the climate, altitude the city, or the soil nature. The soil is heterogeneous, transferable, and provides valuable geographic information [68], and could be useful in human trafficking or movement of a corpse. However, some limitations are noted due to individual lifestyle, travel, or health [22]. Soil adheres to and can be recovered from many surfaces. It could be used to determine the origin of an unknown sample, to limit a search area, or to compare samples from a suspect with those from a crime scene [68,69]. Soils are already studied by their chemical and mineral composition such as type, color, and size, but also by their organic composition [68]. Soil analyses can include palynology, the study of pollen grains and spores, to establish a pollen profile characteristic of a place [14]. These techniques may not be sufficiently discriminative at present, but can be combined in the study of microbial communities.

Soil contains a high quantity of microorganisms that live and interact with each other, representing a unique characteristic of the studied place. Microbial communities show various compositions in different soil types due to different parameters such as concentration in organic or mineral matters, soil alkalinity, pH, and climatic changes [68]. In the MiSAFE project, Demanèche et al. evaluated two metagenomic approaches in forensics. A mock crime scene with disturbed ground-to-hide drugs proved to be distinguishable between two situations, one very close to the mock crime scene (about 25 m) and another about 1 km from the suspect’s home. This study demonstrated that it was possible to detect some variations between soils with the taxonomic or functional differences analyses. It could help link one site to another or even to exclude one when the bacterial community is clearly different [14].

Habtom et al. also evaluated the value of determining the soil origin by microbial communities. They recovered some samples from five known research sites spread over 260 km in Israel (two to four different soil types by site). They demonstrated that microbial communities in the same location are more similar than other communities from the same soil type but at a different location. A significant correlation was also observed linking the geographic distance between the soil samples and the microbial communities detected. However, significant differences in bacterial communities from different soil types were noted in each location [68].

## 8. Factors Influencing Microbiome Analysis

### 8.1. Storage Conditions

Dobay et al. evaluated the change of microbiomes of samples from different body sites and fluids (saliva, vaginal fluid, skin, menstrual and peripheral blood, semen) kept at room temperature over 30 days. They observed that microbial communities were specific to body site. Exposure to ambient conditions did not have a significant impact on these bacterial communities and each body site maintained a typical microbial signature [8]. Diez Lopez et al. evaluated other parameters such as the variation of sample substrate, storage, and time conditions (temperature or relative humidity, sample freshness), confirming no variation in microbiome results [70].

The variation of storage time and temperature on the microbiome present on scalp and pubic hairs was also explored. Williams et al. evaluated three classical temperatures of storage in forensic labs: room temperature, +4 °C and −20 °C for 6 weeks. Time and temperatures of storage had no significant influence on microbial communities [71]. 

Song et al. also evaluated the influence of conservation methods on the fecal microbiome with an immediate extraction, after different temperatures of storage (from−20 °C to ambient temperature) and with the use or not of a preservative. The different storage temperatures did not have a significant influence on the fecal microbiome, however, the immediate freezing with the use of a preservative (ethanol) showed the least impact on the composition and the diversity of bacterial communities [72].

### 8.2. Sampling and Lab Protocol

Hannsen et al. studied the potential impact of sampling and laboratory protocols on the detection and identification of saliva deposited on human skin [7]. Three sampling techniques were evaluated: cotton swab, synthetic swab, and tape. Samples consisted of pure saliva from mouth, saliva deposited between fingers, and samples recovered directly on fingers. Cotton swabs (commonly used in forensic investigation) were less efficient than synthetic swabs or tapes. Bacterial communities from the mouth were more concentrated than those recovered from the skin [7,73]. The bacterial diversity was greater by recovering with tape. More bacterial variation could be noted in skin samples than in saliva samples. Oral, vaginal, and fecal samples were easily distinguished, whereas nasal samples were confused with skin samples [73]. Saliva samples deposited on skin as an intermediate combined with the skin microbiome, confusing the interpretation. Inter-individual variations were observed due to lifestyle or health, but without the influence of the sampling method. Other factors could also influence the results such as the extraction method, the sampling body site, or the library preparation steps. The use of standardized sampling methods and protocols is needed [7]. 

Although the sampling method had no direct impact on the analysis, its role on the quantity of recovered microbial communities was evaluated. Four sampling methods used to evaluate the skin microbiome were compared (swabbing, scraping, glove juice sampling, and punch biopsy). Glove juice was the most powerful method because it detected bacteria localized in the lower layers of the skin with a less aggressive method than biopsy, and it was more stable over time, compared to swabs which recovered more pathogens but with high contamination [34,74]. The time between deposit and collection is important because it could be difficult to accurately identify individual microbial communities, especially because the sampling at a given time is not necessarily representative of the initial microbiota [75]. 

Finally, Chase et al. highlighted the influence of sequencing run on the quality of results. Numerous differences of microbial composition were observed depending on the run, probably due to low-abundance taxa. Thus, the quality of the sampling, its redundancy, and the technical replicates increase the quality of the results [76].

### 8.3. Influence of Hand Washing

Tims et al. compared microbial communities before and after hand washing with hospital soap and air drying. Despite differences between participants, the cutaneous microbiome of each remained similar after washing. Possible contaminations could be detected due to microbial transfer from the environment. Interestingly, in this study, the analysis of cutaneous samples from different countries showed a limited influence of these geographical locations on the microbial communities [77]. However, Fierer et al. observed contradictory results with an impact on the microbial composition after hand washing [25]. We noted that the main taxa (and notably the detection of the core microbiome) were present and similar before and after washing, but the time between the last washing and the sampling influences the composition and its alteration [25]. Diez Lopez et al. added that wearing gloves before sampling did not have a significant impact on microbial detection [70]. Use of cosmetic products was not studied, but antibiotic use seemed not to have a significant impact on hand microbiome variability [75].

### 8.4. Environmental Conditions

Evaluation of the impact of geographic location, seasonal variation, or indoor conditions showed a constant exchange between human and environmental microbiomes [76]. To determine sources of variation, samples from different offices were analyzed in three different towns over 6 weeks. The location in the office had an impact on microbial composition and diversity (floor samples were richer), whereas the surface material had no significant influence. As noted before, the microbiomes were specific to each city. Naturally, the microbial composition of the offices was influenced by human contacts, especially skin microbiome [76]. 

A study conducted in residential kitchens highlighted their exposure to many diverse bacteria species from humans and food products on a daily basis. This result represents an interesting approach in forensics because it meant that an individual could leave traces of their passage in a room via their bacterial community. Conversely, it also demonstrated that each surface sampled and analyzed could be contaminated by an unrelated bacterial source. Thus, it is important to carefully choose relevant surfaces to sample for forensic investigation [78]. 

Investigations on household surfaces showed that the majority of bacteria recovered corresponded to the owners of the houses. The microbiota was mainly composed of skin species (belonging to nose, human hands, and feet), associated with microorganisms linked to the environment. They were highly stable and specific to each house [35,79]. However, the accuracy of the results decreased with time, confirming that the microbiota degraded after being deposited on a surface, without the intervention of the third party. This degradation of the quality of the results was accelerated in a public space that was subjected to many changes compared to a single-family house [35,79]. Lifestyle, and particularly cohabitation with pets, increased this diversity of the microbiota [42,80]. Similarly, a study was conducted to determine the influence of children or pets on the microbiome detected in houses. Family members shared a high level of bacterial diversity, except for very young children whose microbiota was different. Moreover, human microbial communities were similar to those of their dogs, and a high exchange of microorganisms was observed between the hand microbiota of volunteers and their dogs. These microbial communities had homologies with the mouth and forehead of dogs. Curiously, cohabitation with other types of pets seemed to have no significant impact on the human microbiota [80].

## 9. Microbiota and Real Cases

Very few cases have been documented on the use of microbiome studies in real forensic sciences with a penal issue, because to date the NGS technology must be developed and adapted to forensic studies and few teams used it routinely. Its association to human genetic approaches must be established. 

Two cases were reported by Quaak et al. [81], the first case concerned a house robbery where feces and tissues were found near a car used for the burglary. Forensic analysis of the stained tissue detected an unknown male genetic profile. A suspect involved in many robberies was apprehended with a profile matching the genetic profile found on the tissue. However, the defense argued that this profile could have come from another activity, unrelated to the burglary. A microbial analysis then connected the results of the genetic profile and the fecal samples and the suspect was convicted by the court. The second case was a sexual assault, both anally and vaginally, by a nurse at a hospital. This man was apprehended and samples from his genital area were obtained. Analysis of bacterial populations determined a mixture of fecal and vaginal microbiota present on victims and the accused, allowing conviction by the court [81].

Another investigation concerned a rape of a minor. An object that could have been used in the sexual assault was seized. The samples were DNA mixtures of the suspect’s DNA and the majority victim’s DNA. The microbial analysis revealed that the vaginal bacterial community was highly represented on the object used in the rape [82]. Finally, another example of the use of the microbiome in forensics concerned a case of rape by digital penetration. A swab of the suspect’s finger found a DNA mixture of the suspect’s DNA and the victim’s DNA. The bacterial community was mainly composed of vaginal microbiota, proving the digital penetration [82].

## 10. Conclusions

Microbiome study is a promising tool for forensic sciences, despite certain limitations. The major concerns are the stability, reproducibility, and sensitivity of the bacterial analysis. Microbiome analysis is commonly used in medicine and public health, but its application in forensics is more difficult, especially because of the unknown quantity of bacterial communities in the samples. The storage of samples is also controversial because storage temperature and duration affect the quality of the extraction and the determination of bacterial communities. Another limitation is the temporal variation of the individual microbiome, from one body location to another [22]. To answer these concerns, futures studies must generate guidelines for investigations (e.g., sampling methods, number of samples, storage, procedure limitations, and parameters to monitor and control). It is also essential to highlight which bacterial communities are commonly present on supports and surfaces and which can lead to the contamination of the samples [22]. Sampling requires a sufficient amount of bacterial DNA to determine the microbiome or at least the main phyla or classes of bacteria, which are very specific to fluids or tissues [23]. If the NGS technology (provided for example by Illumina (San Diego, CA, USA), Thermo Scientific (Waltham, MA, USA), Qiagen (Hilden, Germany) or Roche (Basel, Switzerland)) is currently present and used routinely in most forensic laboratories for human genetic investigations, its use in microbiota analysis must be developed, which needs high expertise in bioinformatics analysis. Appropriate kits and new protocols must also be adapted due to the low quantity of bacterial DNA available [29]. The cost of this analysis (between 285 and 500$ per sample) remains relatively high, and involves more investigations to clearly establish the place and the role of this approach. 

Moreover, the results must be adapted for the legal sphere and leave no doubt to the court. The question of evidence is a major issue in criminal proceedings. In French criminal law, judges and juries are free to assess the strength of evidence but their personal conviction is based on an analytical evaluation of it. Evidence then becomes a procedural element that must allow the truth of the research to come through and legitimize a criminal decision. The outcome of the criminal trial depends, among other things, on the existence and quality of this criminal evidence. The quality of the evidence is based on its legality in the criminal trial but also several purely scientific parameters such as its integrity, accuracy, and reliability. The admissibility of the results is therefore conditioned by the respect of certain conditions and in particular the carrying out of the analyses by authorized and qualified persons [83]. 

At a crime scene, there are more and more exploitable biological traces, but they are not necessarily related to the crime. The scientific result is nothing without an evaluation of the relevance of this trace. Novel methods of classification will be necessary to use microbial data in forensics, although limitations remain (e.g., human microbiome diversity, data errors in classification, environmental variations, and genetic factors). Microbiological forensic analysis is performed by comparing evidence samples and a known microorganism. To use the microbiome as proof, robust statistical methods have to be validated to produce accurate results, in particular to take into account the different parameters influencing the microbiome composition [22]. Another factor to consider for the future use of the microbiome in forensic sciences is that these studies give information about individual health and lifestyle. It will therefore be essential to define a legal framework for the use of those bacterial data, as with genetic markers in DNA analysis, which are defined by the criminal procedure code.
